# Identification of Ferroptosis-Related Biomarkers for Prognosis and Immunotherapy in Patients With Glioma

**DOI:** 10.3389/fcell.2022.817643

**Published:** 2022-01-31

**Authors:** Junfeng Shi, Donglin Lai, Xiaojia Zuo, Dingsheng Liu, Bing Chen, Yanjun Zheng, Changlian Lu, Xuefeng Gu

**Affiliations:** ^1^ Department of Prosthodontics, Shanghai Ninth People’s Hospital, College of Stomatology, Shanghai Jiao Tong University School of Medicine, Shanghai, China; ^2^ Shanghai Engineering Research Center of Advanced Dental Technology and Materials, National Clinical Research Center for Oral Diseases, Shanghai, China; ^3^ Shanghai Key Laboratory of Molecular Imaging, Zhoupu Hospital, Shanghai University of Medicine & Health Sciences, Shanghai, China; ^4^ School of Health Science and Engineering, University of Shanghai for Science and Technology, Shanghai, China; ^5^ Department of Neurosurgery, Affiliated Hospital of Guangdong Medical University, Guangzhou, China; ^6^ School of Pharmacy, Shanghai University of Medicine & Health Sciences, Shanghai, China

**Keywords:** ferroptosis, lncRNA, biomarker, prognosis, immune checkpoint, immunotherapy, glioma

## Abstract

Ferroptosis is a novel type of iron- and ROS-dependent cell death and is involved in various diseases. LncRNAs are involved and play important roles in the occurrence and development of several cancers. However, researches about the role of ferroptosis-related lncRNAs in glioma are relatively rare. Here, we identified nine ferroptosis-related lncRNAs and then constructed a prognostic model by the LASSO and Cox analysis. The model could predict overall survival with high sensitivity and specificity according to ROC curves. In addition, the cell cycle, p53 signaling, apoptosis, and oxidative phosphorylation pathways were obviously enriched in the pathogenesis of glioma by gene set enrichment analysis. A nomogram was constructed by integrating several independent prognostic clinicopathological features, and it could provide a valuable predictive tool for overall survival. Furthermore, a strong correlation between these nine lncRNAs and immunotherapy was found. Glioma patients in the high-risk group had higher TMB using somatic mutation data, different immune infiltration, and higher expression of immune checkpoints, indicating these patients might benefit from immune checkpoint inhibitor therapy. In summary, these nine ferroptosis-related lncRNAs were promising biomarkers for predicting overall survival and guiding immunotherapy or future immune checkpoint inhibitor development for glioma patients.

## Introduction

Glioma is the most common primary malignant tumor of the central nervous system in adults, accounting for approximately 80% of cases (Stupp et al., 2015; Ostrom et al., 2020). According to the 2016 classification standards of the World Health Organization (WHO), the pathological types of glioma can be divided into low-grade glioma (LGG, grade 1–2) and high-grade glioma (HGG, grade 3–4) (Louis et al., 2016). LGG has high differentiation and a good prognosis, and the median overall survival (OS) is 8–10 years. However, HGG has low differentiation, high malignancy, and poor prognosis. Among the types of HGG, the median OS of anaplastic glioma (WHO grade 3) is 3–4 years, and the prognosis of glioblastoma (GBM, WHO grade 4) is the worst, with a median OS of only 14.6–17 months (Smoll et al., 2013; Stupp et al., 2015; Gu et al., 2019; Litak et al., 2019). Glioma is prone to immune infiltration and recurrence after surgical resection, and there are limited therapeutic options to date. Patients with glioma suffer from a high mortality rate and poor quality of life. In recent years, it has been reported that immune checkpoint inhibitors (ICIs, i.e., PD-L1 inhibitors) have a certain effect in the treatment of glioma (Ampudia-Mesias et al., 2021).

Ferroptosis is a novel form of programmed cell death that is different from apoptosis and autophagy; ferroptosis mainly occurs through Fe^2+^ or lipoxygenases, which catalyse the lipid peroxidation of unsaturated fatty acids that are highly expressed on the cell membrane, thereby inducing cell death (Dixon 2017; Woo et al., 2020; Tang et al., 2021). The level of reactive oxygen species (ROS) in tumor cells is higher than that in normal cells, and excessive accumulation of ROS activates the apoptotic pathway and promotes the death of tumor cells (Wang et al., 2016; Zhao et al., 2019). Iron overload can result in ferroptosis, which can be activated in cancer cells to fight cancer (Brown et al., 2020). And inhibiting ferroptosis results in decreased chemo-sensitivity (Zhang et al., 2020). The application of ferroptosis inducers can enhance tumor sensitivity to chemotherapy and radiotherapy, providing a more promising therapeutic strategy for killing drug-resistant cancer cells (Hassannia et al., 2019; Zhao et al., 2020). The most interesting finding was that the ferroptosis inducer erastin can enhance the sensitivity of GBM cells to temozolomide (Chen et al., 2015).

With the improvement of gene sequencing technology, long noncoding RNAs (lncRNAs) have attracted increasing attention because they can regulate gene expression at multiple levels, such as the chromatin, transcription, and posttranscriptional levels, and they can participate in various biological processes, such as cell differentiation, cell cycle regulation, and stem cell pluripotency maintenance (Lu Q. et al., 2020; Zhuo et al., 2020; Jiang, et al., 2021). In recent years, multiple studies have confirmed that regulation of the expression of lncRNAs (i.e., *LINC00618, LINC00336, PVT1*, and *ZFAS1*) is closely related to ferroptosis (Wang et al., 2019; Lu J. et al., 2020; Yang et al., 2020; Wang et al., 2021; Yao, et al., 2021; Zhang et al., 2021). Some lncRNAs (i.e., *LINC00336* and *ZFAS1*) also act as competitive endogenous RNAs (ceRNAs) to prevent peroxidation, thereby inhibiting ferroptosis (Wang et al., 2019; Yang et al., 2020).

Nevertheless, researches about the potential mechanism of ferroptosis-related lncRNAs in glioma are relatively rare. Herein, nine ferroptosis-related lncRNAs (*AC062021.1, FAM66C, MIR497HG, TMEM72-AS1, AC010729.2, FAM225B, HOXA-AS2, LINC00662, and LINC00665*) were identified to construct a risk model in patients with glioma. This risk model aimed to reveal the potential roles of these biomarkers in the prognosis and treatment prospects of glioma patients and to further explore the relationships among tumor mutational burden (TMB), immune checkpoints, and ferroptosis-related lncRNAs. In particular, the extra application of these biomarkers in glioma patients during treatment with immune checkpoint inhibitors (ICIs) was also explored.

## Materials and Methods

### Data Acquisition

**FIGURE 1:**
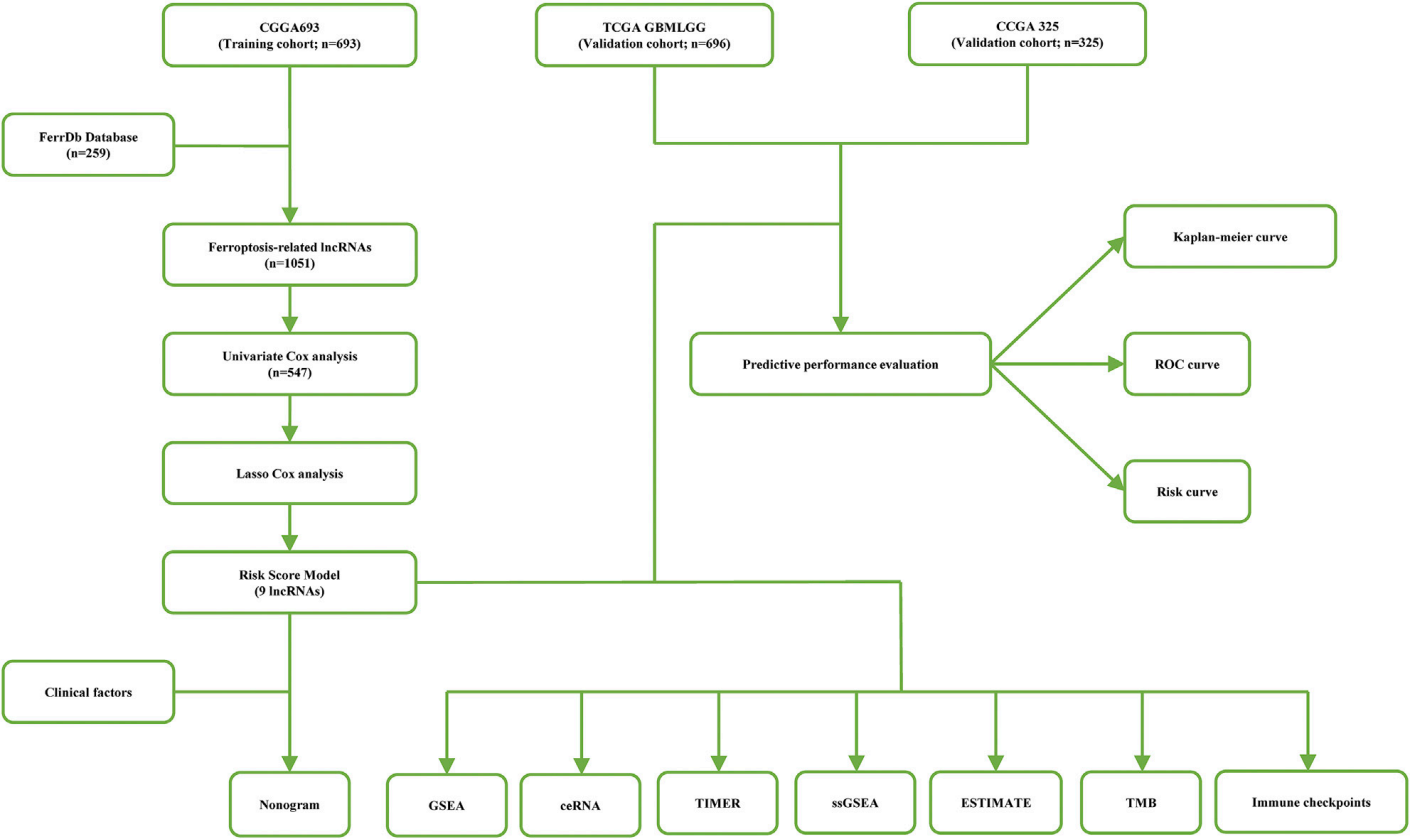
A flow chart of data analysis methods and processing in this study.

A flow chart of the data analysis and processing methods used in this study is shown in Figure 1. Patient data were collected from the Chinese Glioma Genome Atlas (CGGA) (Zhao, et al., 2021) and The Cancer Genome Atlas (TCGA). After excluding samples without complete clinical data, a total of 1714 samples were obtained for final analysis, including the training CGGA693 cohort (*n* = 693), the validation CGGA325 cohort (*n* = 325), and the validation TCGA cohort (LGG, *n* = 528; GBM, *n* = 168). Two hundred fifty-nine ferroptosis-related genes were obtained from the FerrDb database (Supplementary Table S1) (Zhou and Bao 2020).

### Construction of a Prognostic Model

The 259 ferroptosis-related genes and lncRNAs were analyzed in the training CGGA693 cohort by Spearman analysis, and thus, 1051 ferroptosis-related lncRNAs were obtained under the “absolute value of correlation coefficient≥0.3, *p* < 0.05” screening condition. Next, 547 prognosis-related lncRNAs were acquired by univariate Cox regression analysis with the “*p* < 0.05”; screening condition. Then, the least absolute shrinkage and selection operator (LASSO) regression analysis was performed for variable screening and dimension reduction to build a simplified and accurate model. The best lambda and corresponding variables were usually obtained with the least mean square. Lambda. min was 0.09938703, and 24 ferroptosis-related lncRNAs were identified. Multivariate Cox regression analysis was further performed, and 9 ferroptosis-related lncRNAs were ultimately identified to establish a more stable prognostic risk model. The risk score was calculated with the regression coefficients and expression of genes. A Sankey diagram was constructed to visualize the relationship between the 259 ferroptosis-related genes and lncRNAs. According to the expression levels of ferroptosis-related lncRNAs, glioma patients were divided into high- and low-expression groups in the CGGA693 cohort, and then, Kaplan-Meier curves of the two groups were constructed to further explore the value of these 9 ferroptosis-related lncRNA prognostic biomarkers.

### Assessment and Validation

According to the median risk score, glioma patients in the training CGGA693 cohort were divided into high- and low-risk groups. Kaplan-Meier analysis and the long-rank test were applied to identify whether the OS rates of the two risk groups were significantly different. ROC curves (AUCs for 1-, 3-, and 5-years OS) were used to assess the specificity and sensitivity of the predictive model, which could be analyzed by using the “survivalROC” R package. A risk score map, survival status distribution map, and expression heatmap were plotted to display the distribution of patients and the expression of biomarkers between the low- and high-risk groups. To verify whether the risk model was reliable for predicting OS, the same methods were performed in two external cohorts (CGGA325 and TCGA).

### Association of Prognostic Biomarkers and Clinicopathologic Features

To explore the relationship between ferroptosis-related prognostic lncRNAs and clinicopathologic factors, the expression of prognostic biomarkers among subgroups with different statuses was analyzed, including risk stratification, WHO grade, primary or recurrent type, IDH mutation status, and 1p19q codeletion status. These statistical analyses were applied to both the training and validation cohorts.

### Functional Enrichment Analysis

To elucidate the underlying biological mechanism of the present results, gene set enrichment analysis (GSEA) was performed in GSEA V4.1.0 based on the Molecular Signatures Database v7.4 (Subramanian et al., 2005). To explore the regulatory mechanism of the screened candidate lncRNAs, the relationships among miRNAs, mRNAs, and lncRNAs were evaluated, and a potential ceRNA network in the regulation of glioma was constructed. These 9 biomarkers were input into the DIANA tool LncBase v.2 to predict the corresponding miRNAs (Paraskevopoulou et al., 2016). Then, mRNAs targeted by miRNAs were predicted with the condition of no less than three target-predicting programs in the Encyclopedia of RNA Interactomes (Li, et al., 2014). The differentially expressed mRNAs (DEmRNAs) between the high- and low-risk groups were identified by “limma” R. The final mRNAs were identified by integrating predictive mRNAs and DEmRNAs. The ceRNA network was constructed and visualized by Cytoscape (version: v3.7.1) (Shannon et al., 2003). Afterward, the functional enrichment results of these mRNAs in the ceRNA network were obtained by gene ontology (GO), including the biological process (BP), cellular component (CC), molecular function (MF), and Kyoto Encyclopedia of Genes and Genomes (KEGG) pathway analysis via the “clusterProfiler” R package. The p cut-off-value and q cut-off-value were both 0.05.

### Nomogram Construction and Assessment

Univariate and multivariate Cox regression analyses of clinicopathological features were used to identify independent prognostic factors. Then, a nomogram was constructed based on these independent prognostic factors. This nomogram could calculate the OS rates of glioma patients at 1, 3, and 5 years, which provides valuable suggestions for clinicians to judge the OS of glioma patients. In addition, the concordance index (C-index) was calculated to evaluate the predictive ability of a nomogram, and calibration curves were used to evaluate the accuracy of the nomogram. The higher the C-index is, the more accurate the prediction model. Calibration curves, including 1-, 3-, and 5-years curves, were drawn in the “rms” R package to assess whether there was a good match between the actual probability of OS and the predicted probability.

### Immune Infiltration

The association between these ferroptosis biomarkers and six types of immune cells (B cells, CD8^+^ T cells, CD4^+^ T cells, neutrophils, macrophages, and dendritic cells) was identified with the TIMER database (Li T. et al., 2020). Pearson’s correlation coefficients and estimated *p* values were calculated. Subsequently, single-sample GSEA (ssGSEA) was performed to assess the immune response in the two risk groups using the “gsva” R package. Additionally, the ssGSEA scores of 13 immune cell infiltrate and 16 immune-related functions were calculated in the two risk groups and displayed as a heatmap and boxplots. The immune difference between the two risk groups was depicted in the heatmap and violin plots. In addition, stromal scores and immune scores for two risk groups were calculated and compared according to the ESTIMATE algorithm (Yoshihara, et al., 2013).

### Tumor Mutational Burden and Immune Checkpoints

Tumor cells with many mutations were far different from normal cells and thus more easily found by the immune system. Thus, patients with higher TMB theoretically benefit more from immunotherapy. Mutation data acquired by Mutect software were downloaded from the TCGA database, and TMB was calculated using R software. TMB values were analyzed between high- and low-risk groups in the TCGA cohort. Based on the median TMB value, glioma patients were divided into low- and high-TMB groups. In the two TMB groups, survival analysis and correlation analysis between TMB and ferroptosis-related biomarkers were conducted. Moreover, the expression levels of immune checkpoints were compared between the two risk groups.

### Statistical Analysis

The data were statistically processed by the R package. The Wilcox test was used to compare two groups in boxplots. Survival differences were determined by the Kaplan-Meier curve and long-rank test. *p* < 0.05 was considered to indicate statistically significant differences.

## Results

### Construction of a Prognostic Model

**FIGURE 2:**
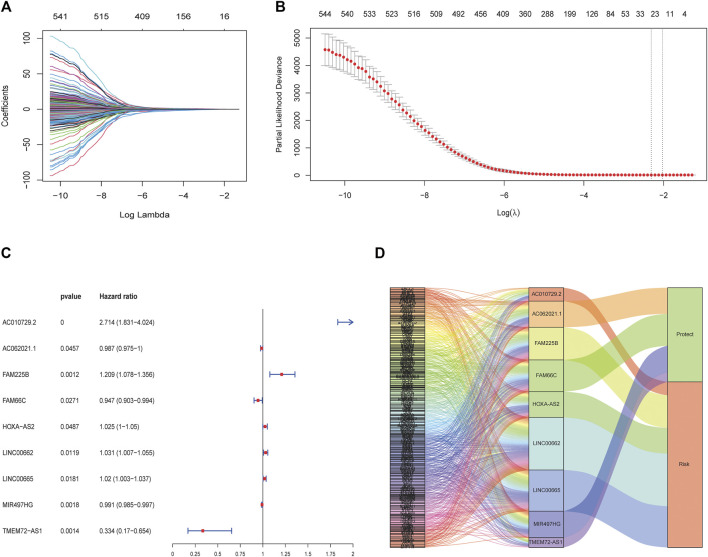
Construction of the ferroptosis-related lncRNA prognostic biomarkers. **(A, B)** LASSO regression model. The red dots indicate the partial likelihood of deviance values, the grey lines indicate the standard error (SE), and the two vertical dashed lines on the left and right indicate optimal values by minimum criteria and 1-SE criteria, respectively. **(C)** Multivariate Cox regression analysis revealed that 9 ferroptosis-related lncRNAs were independent prognostic factors for glioma patients. **(D)** Sankey diagram shows the relationship among ferroptosis-related genes, ferroptosis-related lncRNAs, and risk types.

**FIGURE 3:**
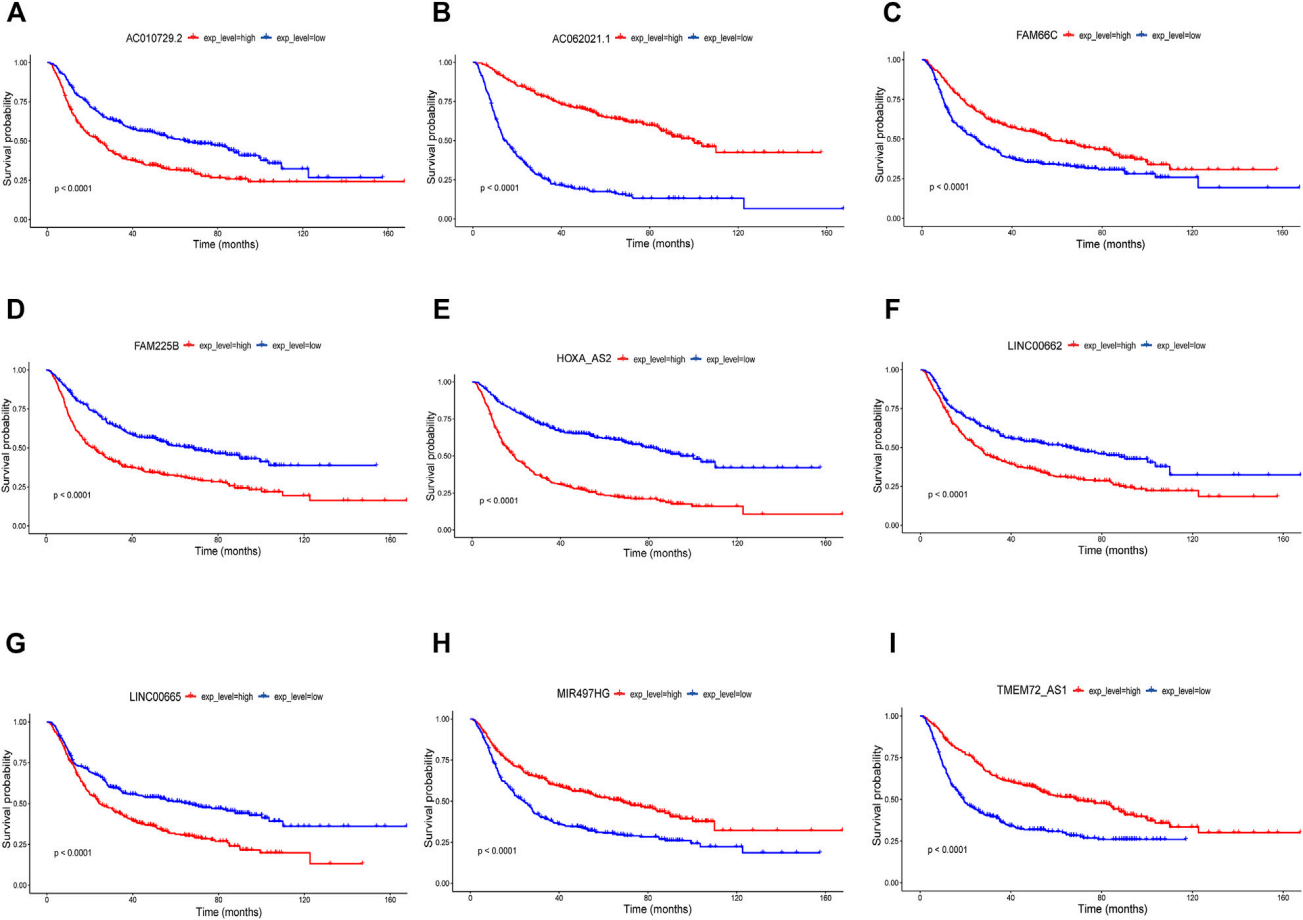
Nine biomarkers in Kaplan-Meier analysis in the training CGGA693 cohort **(A)** AC010729.2, **(B)** AC062021.1, **(C)** FAM66C, **(D)** FAM225B, **(E)** HOXA-AS2, **(F)** LINC00662, **(G)** LINC00665, **(H)** MIR497HG and **(I)** TMEM72-AS1.

The clinical information of the glioma patients obtained from the CGGA and TCGA cohorts is summarized in Table 1 and Table 2. Nine ferroptosis-related prognostic lncRNAs were identified in the training CGGA693 cohort after serial analysis, including Spearman analysis, univariate Cox regression analysis, and LASSO-Cox regression analysis (Figures 2A–C). The risk score was thus calculated by the expression of 9 ferroptosis-related lncRNAs and their corresponding regression coefficients: Risk score = 0.9849102 * Exp_AC010729.2_–0.01261145 * Exp_AC062021.1_ + 0.18957587 * Exp_FAM225B_—0.05426328 * Exp_FAM66C_ + 0.02465331 * Exp_HOXA-AS2_ + 0.03022672 * Exp_LINC00662_ + 0.01995719 * Exp_LINC00665_–0.00924465 * Exp_MIR497HG_—1.09779996 * Exp_TME72-AS1_ (Supplementary Table S2). These lncRNAs included 4 protective biomarkers (*AC062021.1, FAM66C, MIR497HG, and TMEM72-AS1*) and 5 risk biomarkers (*AC010729.2, FAM225B, HOXA-AS2, LINC00662, and LINC00665*). A Sankey diagram was drawn to visualize the association among these 259 ferroptosis-related genes, 9 ferroptosis-related lncRNAs, and OS in patients with glioma (Figure 2D). Furthermore, survival analysis of these biomarkers in the training CGGA693 cohort was conducted (Figure 3).

**TABLE 1:** The clinical characteristics of glioma patients in CGGA.

Characteristics	Training	Validation 1
	CGGA693 (*n* = 693)	CGGA325 (*n* = 325)
PRS type		
Primary	422	229
Recurrent	271	62
Secondary	0	30
Grade		
WHO II	188	103
WHO III	255	79
WHO IV	249	139
Gender		
Female	295	122
Male	398	203
Age		
≥ 60	83	33
<60	609	292
Follow-up state		
Alive	266	96
Dead	397	220
Radiotherapy		
Untreated	136	66
Treated	510	244
Chemotherapy		
Untreated	161	111
Treated	486	193
IDH mutation status		
Mutant	356	175
Wildtype	286	149
1p19q codeletion status		
Non-codel	478	250
Codel	145	67
MGMTp methylation status		
Methylated	315	157
Un-methylated	227	149

**TABLE 2:** The clinical characteristics of glioma patients in TCGA.

Characteristics	Validation 2
	TCGA (*n* = 696)
Type	
GBM	168
LGG	528
Follow-up state	
Alive	420
Dead	273
Gender	
Female	298
Male	398
Age	
≥ 60	158
<60	538

### Assessment and Validation

**FIGURE 4:**
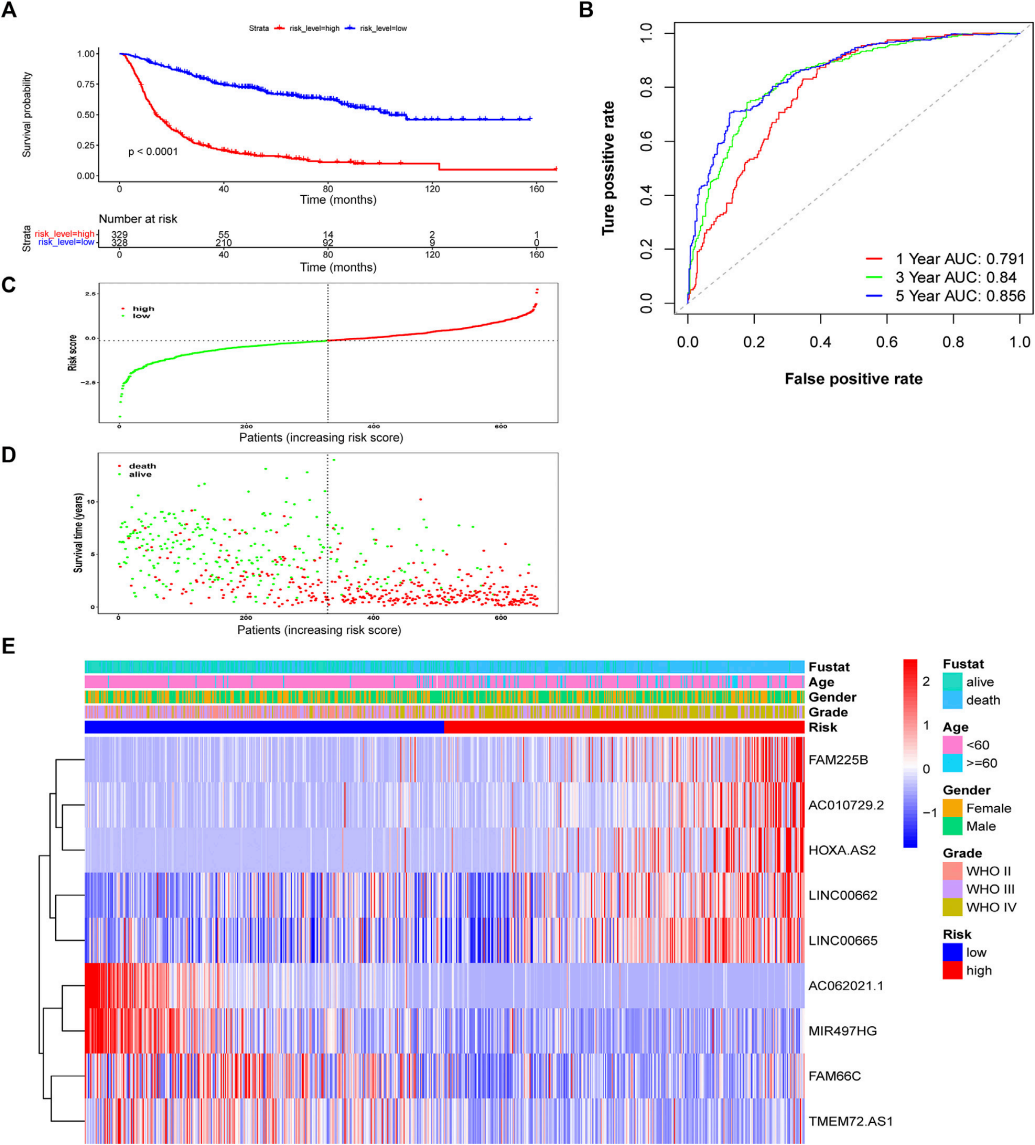
Assessment of the risk score model based on ferroptosis-related prognostic biomarkers in the training CGGA693 cohort. **(A)** Kaplan-Meier survival based on risk level. **(B)** ROC curves for predicting OS of glioma patients based on the risk score. **(C–E)** The risk score, survival distribution of patients with increased risk scores, and expression heatmap of ferroptosis-related lncRNAs based on risk level.

According to the median risk score, patients in the training CGGA693 cohort were divided into two groups. The survival analysis showed that the OS (median OS: 94.5 months) of the low-risk group was significantly prolonged compared with that (median OS: 22.2 months) of the high-risk group (Figure 4A, *p* < 0.0001). To assess the specificity and sensitivity of this prognostic risk model in glioma, time-independent ROC curves were plotted, and the AUCs used to predict the 1-, 3-, and 5-years OS of glioma patients were 0.791, 0.84, and 0.856, respectively (Figure 4B). The risk score curve, the survival status curve, and the expression heatmap of these prognostic biomarkers in glioma patients are shown in Figures 4C–E. The survival status curve showed a larger proportion of dead patients in the high-risk group, which was consistent with the survival analysis. The results were validated in the CGGA325 and TCGA cohorts (Supplementary Figures S1, S2).

### Association of Prognostic Biomarkers and Clinicopathologic Features

**FIGURE 5:**
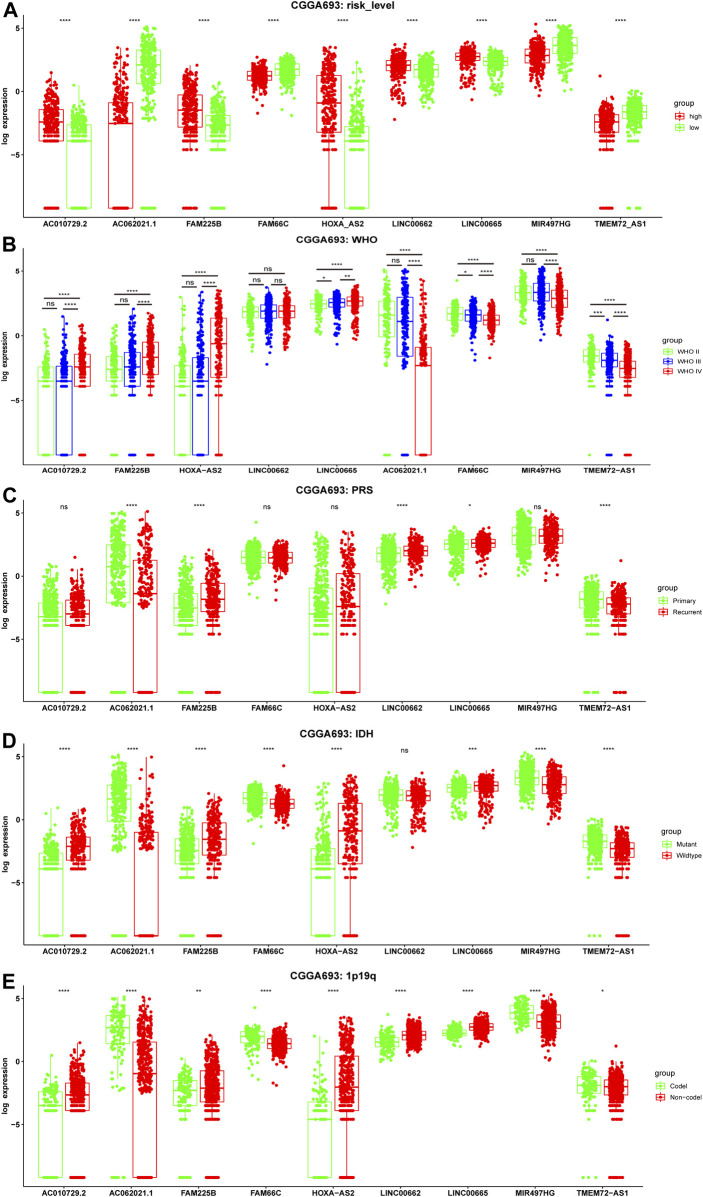
Correlation between the expression level of nine ferroptosis-related lncRNAs and clinicopathological features in the training CGGA693 cohort. **(A–E)** Risk level, WHO grade, PRS type, IDH mutation status, and 1p19q codeletion status. WHO: World Health Organization; P: Primary, R: Recurrent, S: Secondary Recurrent; ns: Not significant, *p < 0.05, **p < 0.01, ***p < 0.001.

To determine whether these ferroptosis-related prognostic lncRNAs were associated with clinical features, expression in different clinical groups was analyzed. There were significant differences in the expression of 9 ferroptosis-related prognostic lncRNAs between the high- and low-risk groups in the CGGA693 cohort (Figure 5A), which were validated in the CGGA325 cohort (Supplementary Figure S3A) and the TCGA cohort (Supplementary Figure S4A). Compared with the WHO grade II group, the expression of *FAM66C* and *TMEM72-AS1* was significantly different in the WHO grade III group, and the expression of eight prognostic biomarkers was significantly different in the WHO grade IV groups (CGGA693 and CGGA325 cohorts) (Figure 5B, Supplementary Figure S3B). Compared with the primary glioma group, the expression of *AC062021.1, FAM225B, LINC00662, LINC00665*, and *TMEM72-AS1* was significantly different in the recurrent glioma group of the training CGGA693 cohort (Figure 5C). However, only *AC062021.1, HOXA-AS2*, and *TMEM72-AS1* in the recurrent glioma group were significantly different, and only *AC062021.1, FAM66C,* and *LINC00662* were significantly different in the secondary recurrent glioma group of the validation CGGA325 cohort, which may be caused by the limited sample size (Supplementary Figure S3C). There were different expression levels of eight ferroptosis-related prognostic lncRNAs between the groups with and without IDH gene mutations in the CGGA693 cohort (Figure 5D), which was consistent with the CGGA325 cohort (Supplementary Figure S3D). Similar findings were demonstrated between the two types of groups with and without 1p19q codeletion (Figure 5E), which was validated in the CGGA325 cohort (Supplementary Figure S3E). In addition, the expression of all 9 lncRNAs was significantly different between the GBM and LGG subgroups in TCGA cohort (Supplementary Figure S4B).

### Functional Enrichment Analyses

**FIGURE 6:**
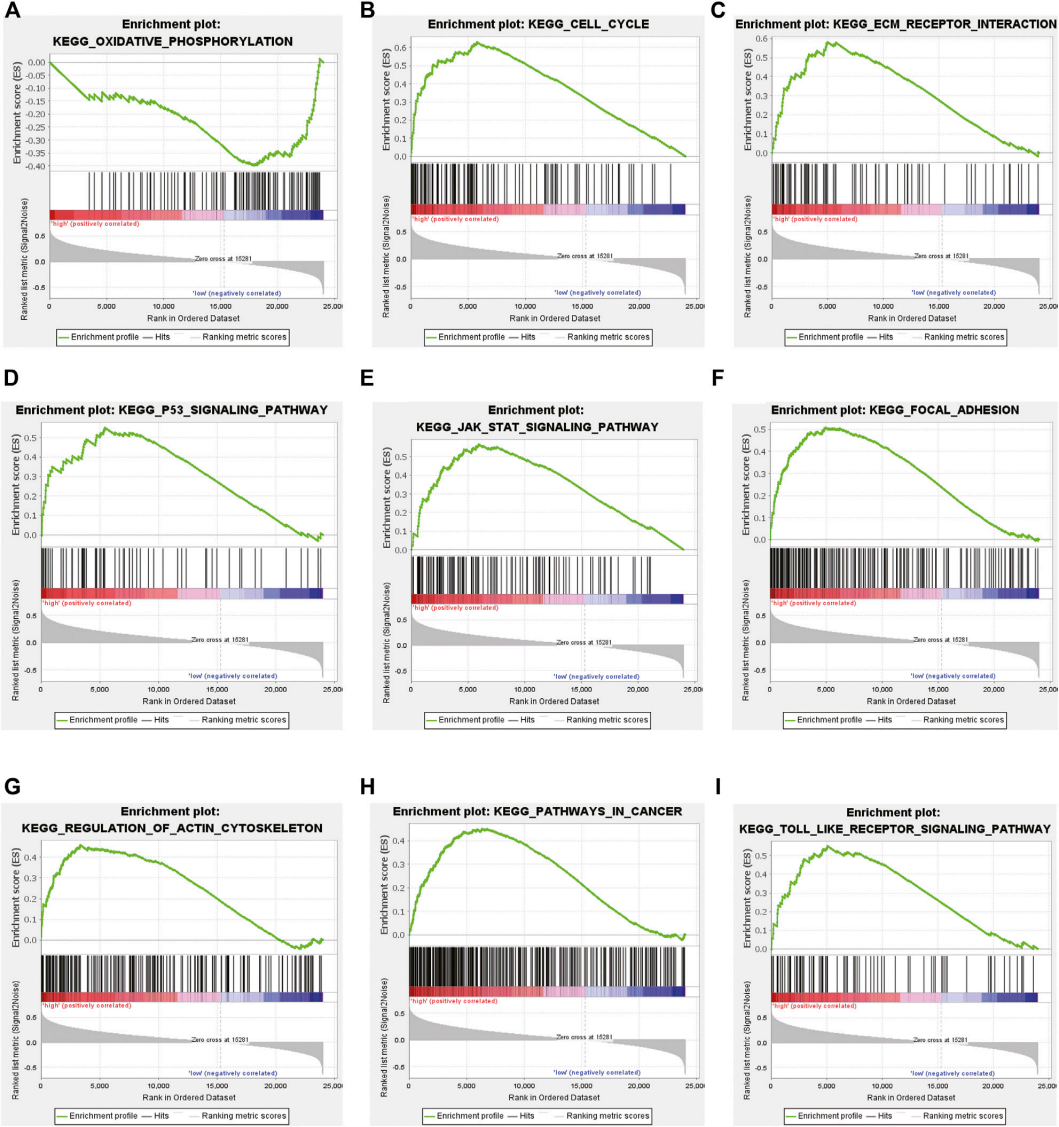
Gene set enrichment analysis (GSEA) in the training CGGA693 cohort. **(A)** GSEA suggested notable enrichment of pathways in the low-risk group and **(B–I)** GSEA suggested significant enrichment in the high-risk group.

To elucidate the underlying biological mechanism of the differences between the two risk groups, GSEA was performed in the CGGA693 cohort. The results showed that oxidative phosphorylation was significantly enriched in the low-risk group (Figure 6A). Several key pathways (cell cycle, ECM-receptor interaction, p53 signaling, JAK-STAT signaling, focal adhesion, regulation of actin cytoskeleton, cancer and Toll-like receptor (TLR) signaling pathways) were significantly enriched in the high-risk group (Figures 6B–I).

**FIGURE 7:**
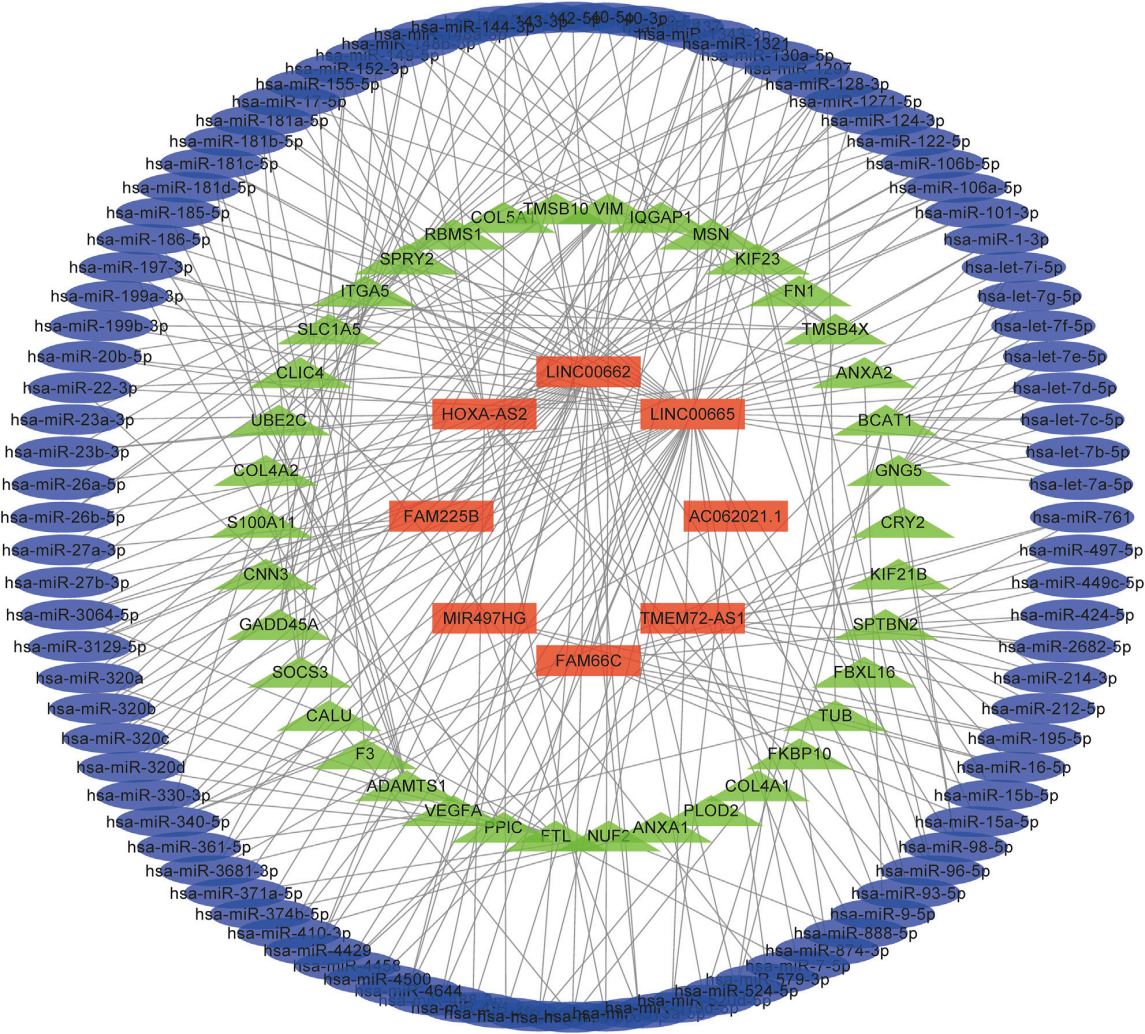
A ceRNA network in the regulation of glioma. Light blue, light green, and red colours represent miRNAs, mRNAs, and lncRNAs, respectively.

The expression correlations of lncRNA-miRNA and miRNA-mRNA in potential ceRNA pairs in glioma were predicted to explore the possible regulatory mechanism of lncRNAs. Only eight of 9 lncRNAs were found to have regulatory mechanisms. (Figure 7, Supplementary Table S3). They may play important roles in the transcriptional regulation of glioma. GO analysis showed that the regulation of angiogenesis was obviously enriched in the BP term of glioma (Supplementary Figure S5). Chen et al. reported that elevated ATF4 expression can enhance proliferation, migration, and angiogenesis in glioma, but the ferroptosis inducer erastin can attenuate this effect. This result implied that ferroptosis could inhibit tumor angiogenesis (Chen, et al., 2017). Moreover, the PI3K-Akt signaling pathway was enriched in the KEGG term of glioma (Supplementary Figure S5). Yi et al. found that activating the mutation of PI3K can result in ferroptosis resistance in tumor cells, while the expression of *SREBP1* or *SCD1* can inhibit the PI3K/Akt/mTOR axis by regulating lipid metabolism, which can sensitize the ferroptosis of cancer cells and play an antitumor role (Yi, et al., 2020).

### Nomogram Construction and Assessment

**FIGURE 8:**
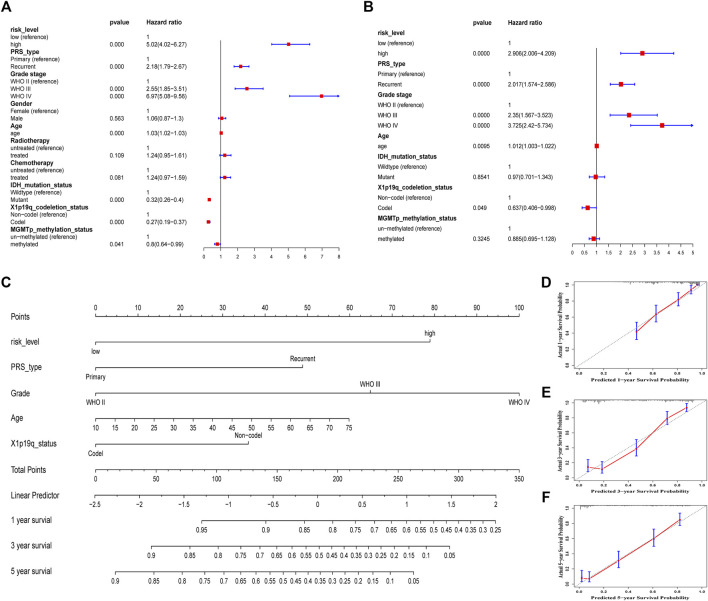
Construction and assessment of a nomogram in the training CGGA693 cohort. **(A)** Univariate Cox regression. **(B)** Multivariate cox regression. **(C)** A prognostic nomogram was used to predict the 1-, 3-, and 5-years survival probability of patients with glioma. **(D–F)** Calibration curves of the nomogram at 1, 3, and 5 years. The *Y*-axis and *X*-axis represent the actual and nomogram-predicted survival probability, respectively.

Some clinical characteristics related to the prognosis of glioma were identified after univariate and multivariate Cox regression analyses in the CGGA693 cohort. To improve predictive ability and provide a quantitative tool to predict the survival outcomes of glioma patients in clinical practice, a nomogram was constructed based on these independent prognostic factors, including risk level, primary or recurrent type, grade, age, and 1p19q codeletion status (Figures 8A–C). The C-index was 0.786, which indicated that this nomogram model had a good predictive value. Calibration curves confirmed again that there was a good match between the actual probability of 1-year, 3-years, and 5-years OS and the predicted probability of the nomogram (Figures 8D–F).

### Immune Infiltration

**FIGURE 9:**
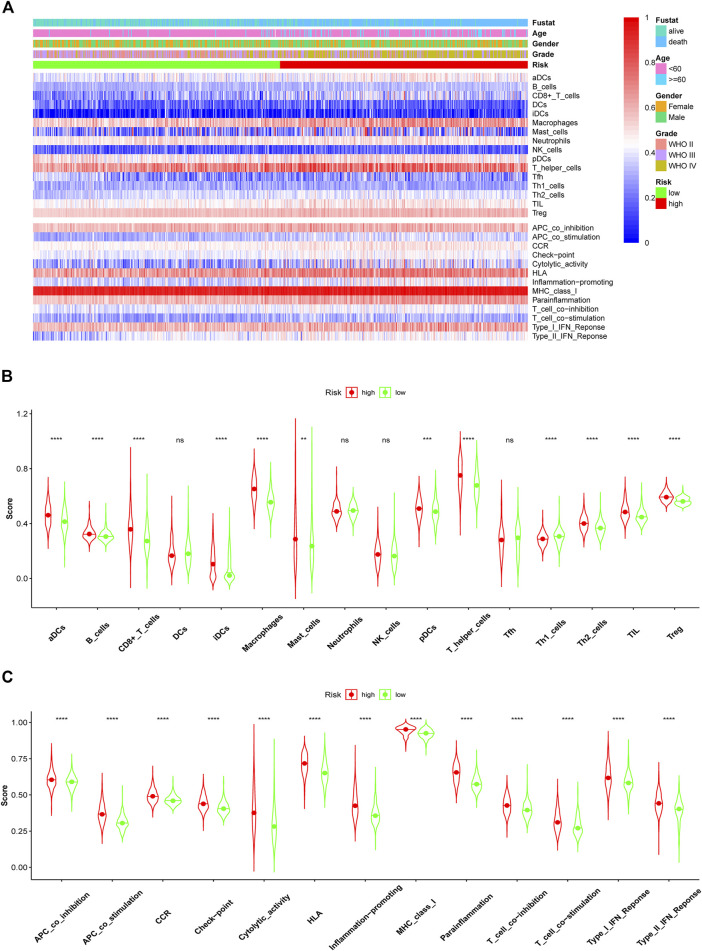
Comparison of immune cell infiltration and functions elevated by ssGSEA between the high- and low-risk groups in the training CGGA693 cohort. **(A)** Heatmap of ssGSEA scores of immune cells and functions. The ssGSEA scores of 16 immune cells **(B)** and 13 immune functions **(C)** between the high- and low-risk groups of glioma patients in violin plots.

**FIGURE 10:**
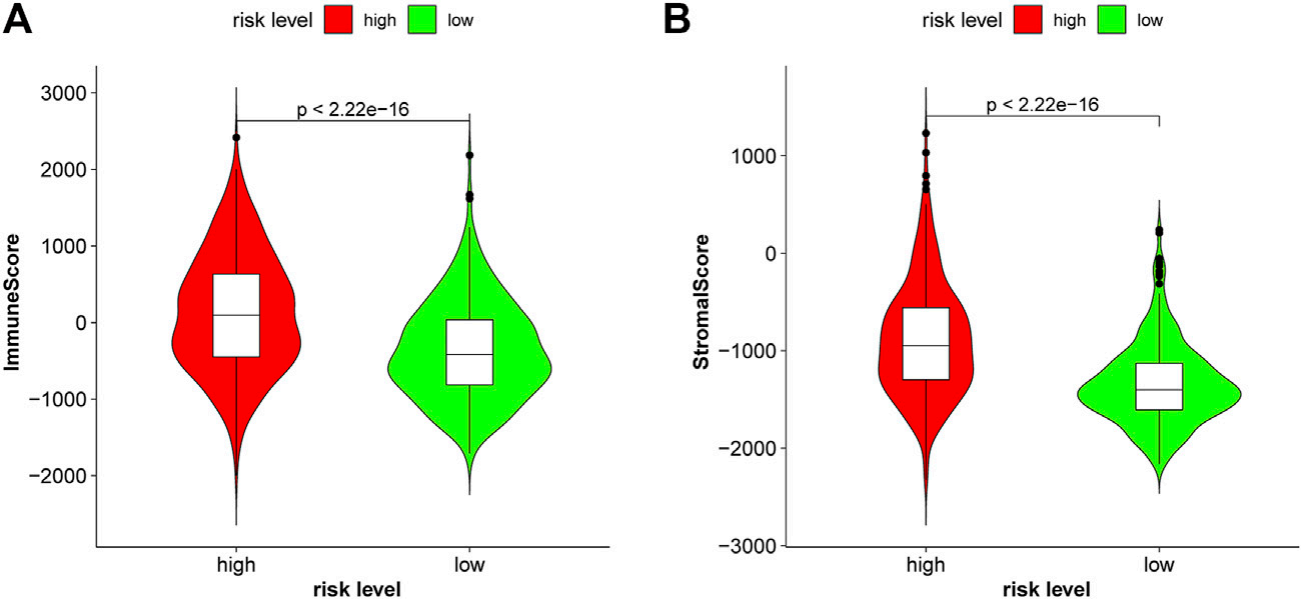
Patients with glioma in the high-risk group had higher immune scores **(A)** and higher stromal scores **(B)** in the CGGA693.

Glioma, especially GBM, with high malignancy and a high recurrence rate, did not respond well to traditional treatment. Here, we studied whether these lncRNAs were associated with immune infiltration and thus could guide their potential application in immunotherapy in glioma. The correlation of *FAM66C* and distinct immune cells was elucidated by the TIMER database (Supplementary Figure S6). In addition, different scores were discovered for several immune cells and immune-related functions in the two risk groups by ssGSEA. The heatmap of the ssGSEA score displayed distinct immune infiltration statuses in the two risk groups of this ferroptosis-related model (Figure 9A). In the Violin diagram, most of the 16 immune cells had different scores between the high- and low-risk groups (Figure 9B). Similar results were also observed for 13 immune-related functions (Figure 9C), especially for immune checkpoints. These results implied that comprehensive analysis of immunological cells and functions may be essential before immunotherapy, especially before ICI therapy. In addition, patients in the high-risk group had higher immune scores and stromal scores (Figure 10).

### Tumor Mutational Burden and Immune Checkpoints

**FIGURE 11:**
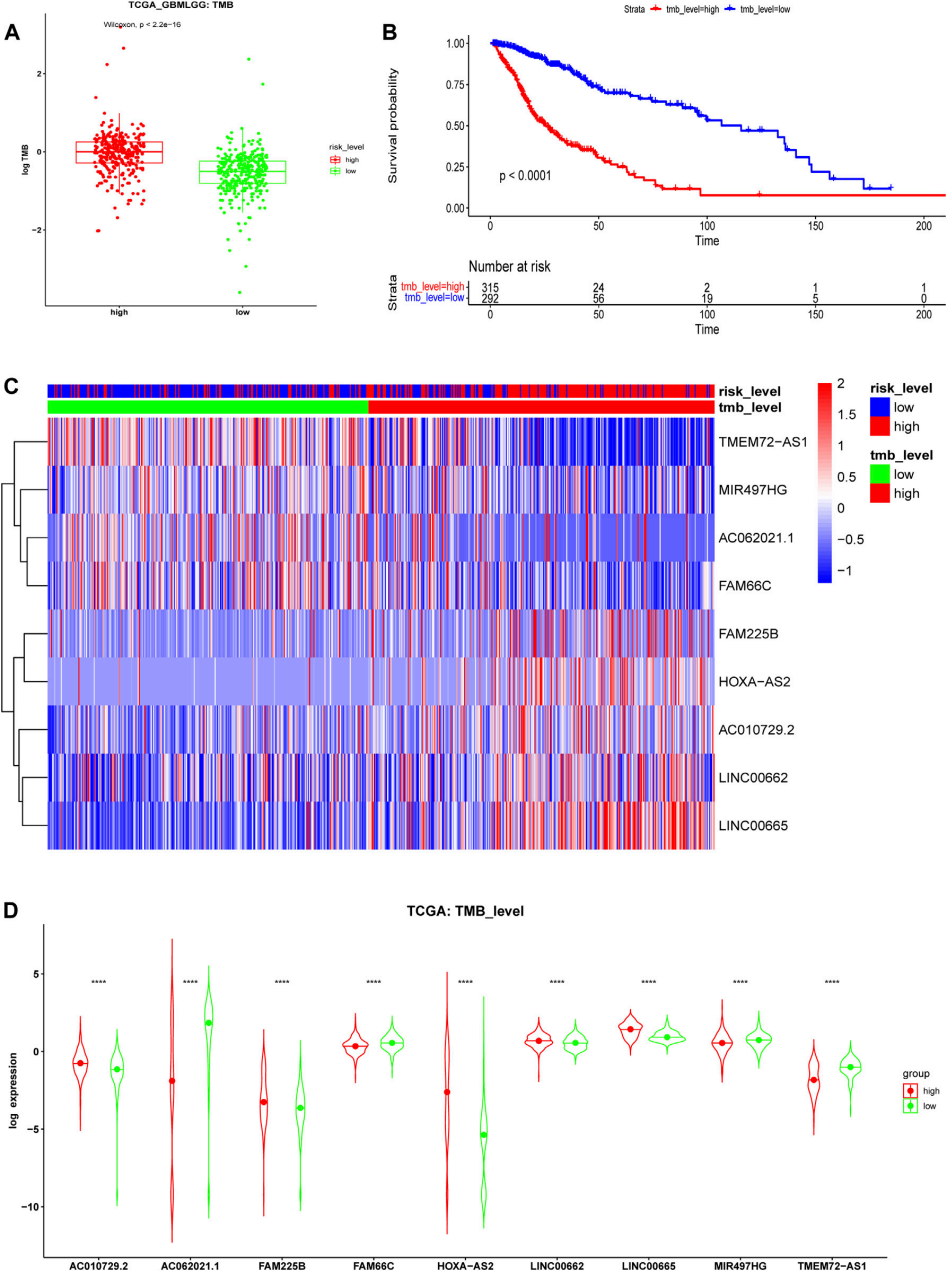
Tumor mutational burden (TMB) analysis in the TCGA cohort. **(A)** TMB levels of high- and low-risk groups. **(B)** Kaplan-Meier survival analysis between high- and low-TMB groups divided by the median TMB. **(C)** Expression heatmap and **(D)** violin plots of 9 ferroptosis-related lncRNAs between the high- and low-level TMB groups in the TCGA cohort.

TMB is a novel biomarker of the response of patients with malignant tumors to ICIs (Ready, et al., 2019). The FDA approved high TMB levels as one of the treatment standards for solid tumor patients to receive medical prescriptions with ICIs. The analysis results of the TCGA cohort showed that the risk stratification calculated from our risk model was consistent with the level of TMB. Patients at high risk had a higher TMB level than those at low risk (Figure 11A, *p* < 0.001), which indicated that glioma patients at high risk may benefit from immunotherapy. Kaplan-Meier curves showed that the OS in the high-TMB group was significantly lower than that in the low-TMB group (Figure 11B, *p* < 0.001). The expression heatmap of these 9 ferroptosis-related prognosis lncRNAs and TMB was shown in Figure 11C. The expression of these nine lncRNAs was significantly different between the high- and low-level TMB groups (Figure 11D), which affirmed that TMB was associated with the lncRNAs in our model.

**FIGURE 12:**
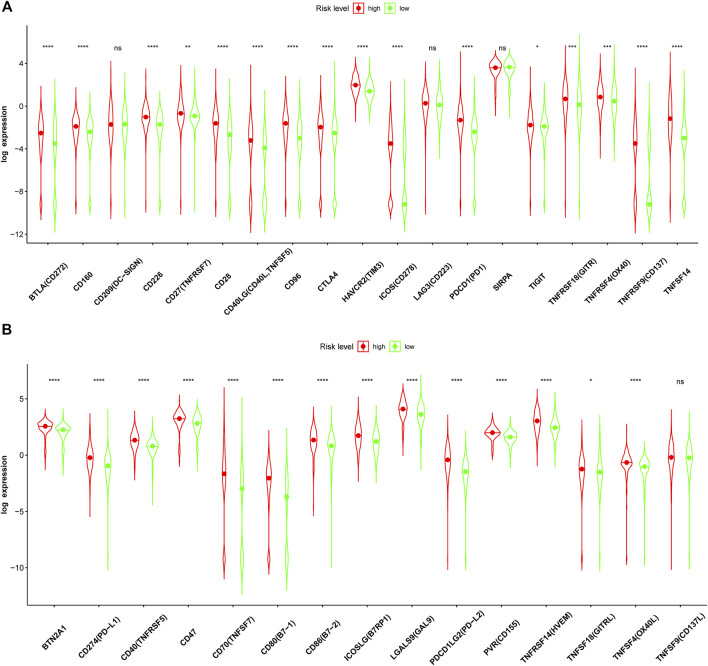
Immune checkpoint analysis in the CGGA693 cohort. **(A, B)** Expression of immune checkpoints between the high- and low-risk groups in the training CGGA693 cohort.

Given the higher ssGSEA score of immune checkpoints and higher TMB in the high-risk group, the expression of immune checkpoints based on the risk level was further analyzed in detail. The results showed that the expression levels of many immune checkpoints in the high-risk group were significantly higher than those in the low-risk group in the CGGA693 cohort (Figure 12). Therefore, glioma patients with high risk in our model might receive more benefits from ICI therapy.

## Discussion

The role of ferroptosis, a new form of cell death, in malignant tumors has been gradually elucidated, and more ferroptosis-related genes have been identified (Liang et al., 2020; Liu et al., 2020; Zhuo et al., 2020). In recent years, ferroptosis-related lncRNAs, which play a regulatory role in protein-coding, have attracted increasing attention (Jiang et al., 2021; Wu and Liu 2021; Zhang et al., 2021). After data mining and analysis, we constructed a ferroptosis-related lncRNA risk model with prognostic value in the CGGA693 cohort and further verified the results in the CGGA325 and TCGA cohorts. In addition, a nomogram was constructed to provide valuable suggestions for clinicians to judge the OS of glioma patients. Based on the TMB level, the immune status and expression of immune checkpoints displayed a strong positive correlation between the two groups in our risk model. Thus, our risk model might be used to guide immunotherapy in patients with glioma.

The dysregulation of 9 ferroptosis-related lncRNAs was related to the OS of glioma patients, especially those with wild-type IDH, 1p19q codeletion, disease recurrence, and tumor malignancy. Some of these lncRNAs have been previously reported to play a role in glioma and other malignant tumors. For example, *FAM225B* plays an important role in cell migration and focal adhesion and is related to the OS of glioma patients (Li, et al., 2020; Ma and Liu 2021). *HOXA-AS2* is involved in the pathogenesis of glioma and regulates glioma cell viability, cell migration, and invasion, participating in the occurrence of vasculogenic mimicry and apoptosis (Gao et al., 2018; Wu et al., 2019). Wu et al. revealed that *LINC00662* in the ceRNA network (the LINC00662/miR-107/HMGB1 axis) regulated cell proliferation and the invasion of glioma and could serve as a therapeutic target for patients with glioma (Wu et al., 2020). More interestingly, *LINC00665* can encode a special micropeptide (CIP2A-BP) to inhibit the progression of three negative breast cancers or act as a ceRNA that is involved in regulating the LINC00665/AGTR1/miR-34a-5p axis and other biological behaviours in glioma (Guo et al., 2020; Ruan et al., 2020; Dai et al., 2021). Additionally, *MIR497HG* was reported to be associated with proliferation, migration, invasion, and lymph node metastasis in bladder cancer and breast cancer (Zhang et al., 2019; Zhuang et al., 2020). However, until now, there have been no reports on the function of these lncRNAs in ferroptosis; therefore, future in-depth work is required.

GSEA showed that oxidative phosphorylation-related genes were significantly enriched in the low-risk group. Oxidative phosphorylation (OXPHOS) and glycolysis can maintain tumor propagation by isogenic glioma stem cells (GSCs) but the former can switch to the latter under metabolic stress (Shibao et al., 2018). In addition, both of them can sustain the emergence of glioma independently by detecting the metabolic requirements of GSCs (Saga et al., 2014; Yoshida and Saya 2021). Gboxin, an oxidative phosphorylation inhibitor, exerts specific toxicity in glioblastoma (Shi et al., 2019). Another p53 signaling pathway enriched in the high-risk group may play an essential role in the ferroptosis-related regulation of glioma. EX527, a Sirt-1 inhibitor, can inhibit the growth of glioma by activating the p53 signaling pathway, which was reported to be related to ferroptosis in lung cancer, and HOXA-AS2 may be associated with the p53 gene in hepatocellular carcinoma (Mao et al., 2018; Lu J. et al., 2020; Lu Q. et al., 2020; Wang et al., 2020; Zhao et al., 2020). Therefore, ferroptosis-related lncRNAs may play an important role in the potential ferroptosis-related regulatory mechanism of glioma.

Several studies have shown that TMB is a very effective prognostic marker and is associated with immune checkpoints (PD-1, PD-L1, etc.) in many cancers, including GBM (Yarchoan et al., 2017; Litak et al., 2019; Ready et al., 2019; Wang and Li 2019; Marabelle et al., 2020; Yin et al., 2020). The FDA approved TMB-H as one of the treatment standards for solid tumor patients to receive ICIs (i.e., a PD-1 inhibitor, pembrolizumab) (Strickler et al., 2021). Our study confirmed that the TMB level predicts the OS of glioma patients (Figure 11B) and is associated with ferroptosis-related biomarkers (Figure 11D). Given the important role of ICIs in diverse cancers (André et al., 2020; Cacciotti et al., 2020; Ott et al., 2020), we further analyzed all immune checkpoints in glioma and found that most of those immune checkpoints, such as PD-1, PD-L1, PD-L2, CTLA4, and TIM3, were significantly different between the two risk groups (Figure 12). Gliomas with a high TMB may benefit from PD-1 inhibitors (Touat et al., 2020). However, gliomas usually have a low TMB and are associated with a highly immunosuppressive microenvironment, which may be a potential mechanism of immunotherapy resistance (Touat et al., 2020). Moreover, ICIs activate the body’s antitumor immune response by blocking immune checkpoints, and the side effects (immune-related adverse events, irAEs) induced by activating the immune system are a major challenge in clinical practice (Postow et al., 2018). Notably, irAEs of immunotherapy have a very high incidence (54–76%), and different ICIs cause different toxic effects (Xu et al., 2018). For instance, nivolumab (anti-PD-1) usually causes endocrine toxicities; atezolizumab (anti-PD-L1) mainly causes hypothyroidism, nausea, and vomiting; pembrolizumab (anti-PD-1) mainly causes arthralgia, pneumonitis, and hepatic toxicities; and iplimumab (anti-CTLA4) mainly causes skin, gastrointestinal, and renal toxicities. Therefore, more ICIs need further exploration to reveal their efficacy and safety. Some ICIs (MEDI6469, tislelizumab, sotigalimab, avelumab, and tremelimumab) of currently ongoing clinical trials in cancers are summarized in Table 3 and have been used in animal studies, clinical trials, or patients with cancer (André et al., 2020; Cacciotti et al., 2020; Herbst et al., 2020; Powles et al., 2020; Armand et al., 2021; Baas et al., 2021; Duhen et al., 2021; Goldman et al., 2021; O'Hara et al., 2021; Ye et al., 2021; Zhai et al., 2021). Some of the immune checkpoints in our study do not currently have corresponding ICIs, suggesting that ICIs could be further developed and may play a crucial role in glioma immunotherapy in the near future.

**TABLE 3:** Currently ongoing clinical trials for immune checkpoint inhibitors in cancer.

Immune checkpoint	Monoclonal antibody	NCT number
CTLA4	Ipilimumab, tremelimumab	NCT04084951
HAVCR2 (TIM3)	Cobolimab (TSR-022), MBG-453, INCAGN02390	NCT04139902, NCT02608268, NCT04370704
PDCD1 (PD1)	Pembrolizumab, nivolumab, tislelizumab, toripalimab, TSR-042	NCT02563002, NCT03113266, NCT03307785
TIGIT	BMS-986207, AMR101, Icosapent Ethyl Oral Capsule	NCT04570839, NCT03661047, NCT04682665
TNFRSF4 (OX40)	PF-04518600	NCT03092856, NCT03971409
TNFRSF9 (CD137)	GVAX	NCT03767582
CD274 (PD-L1)	Atezolizumab, durvalumab, avelumab, IMC-001	NCT04084951, NCT04230759, NCT04268368, NCT04196465
CD40 (TNFRSF5)	APX005M (sotigalimab)	NCT03597282, NCT03719430
CD47	PF-07257876, TTI-621, HX009	NCT04881045, NCT02890368, NCT02663518, NCT04886271
CD80 (B7-1)	IMC-001	NCT04196465

## Conclusion

In summary, we first identified 9 ferroptosis-related lncRNAs that could be independent prognostic biomarkers of glioma patients and created a prognostic risk model of glioma. This risk model based on these nine biomarkers can predict the outcome of patients with glioma in some clinical conditions, such as IDH wildtype, 1p19q codeletion, and disease recurrence; in particular, the nomogram can predict the OS rate of clinical patients, which may provide a valuable suggestion for clinicians to judge the OS of glioma patients. Additionally, this risk model may have potential application value for guiding immunotherapy or future ICI development for glioma patients.

## Data Availability

The original contributions presented in the study are included in the article/Supplementary Material, further inquiries can be directed to the corresponding authors.

## Abbreviations

BP, biological process; CC, cellular component; ceRNAs, competitive endogenous RNAs; CGGA, the Chinese Glioma Genome Atlas; C-index, the concordance index; DEmRNAs, differentially expressed mRNAs; GBM, glioblastoma; GO, gene ontology; GSEA, gene set enrichment analysis; HGG, high-grade glioma; ICIs, immune checkpoint inhibitors; KEGG, Kyoto Encyclopedia of Genes and Genomes; LASSO, least absolute shrinkage and selection operator; LGG, low-grade glioma; lncRNAs, long noncoding RNAs; MF, molecular function; OS, overall survival; ROS, reactive oxygen species; ssGSEA, single-sample GSEA; TCGA, The Cancer Genome Atlas; TIMER, Tumor immune estimation resource; TMB, tumor mutational burden; WHO, the World Health Organization.
