# Supplementary figures and images for: Identification of Ferroptosis-Related Biomarkers for Prognosis and Immunotherapy in Patients With Glioma
Source: Front Cell Dev Biol. 2022 Jan 31;10:817643. doi: 10.3389/fcell.2022.817643 (PMC8842255; doi:10.3389/fcell.2022.817643)

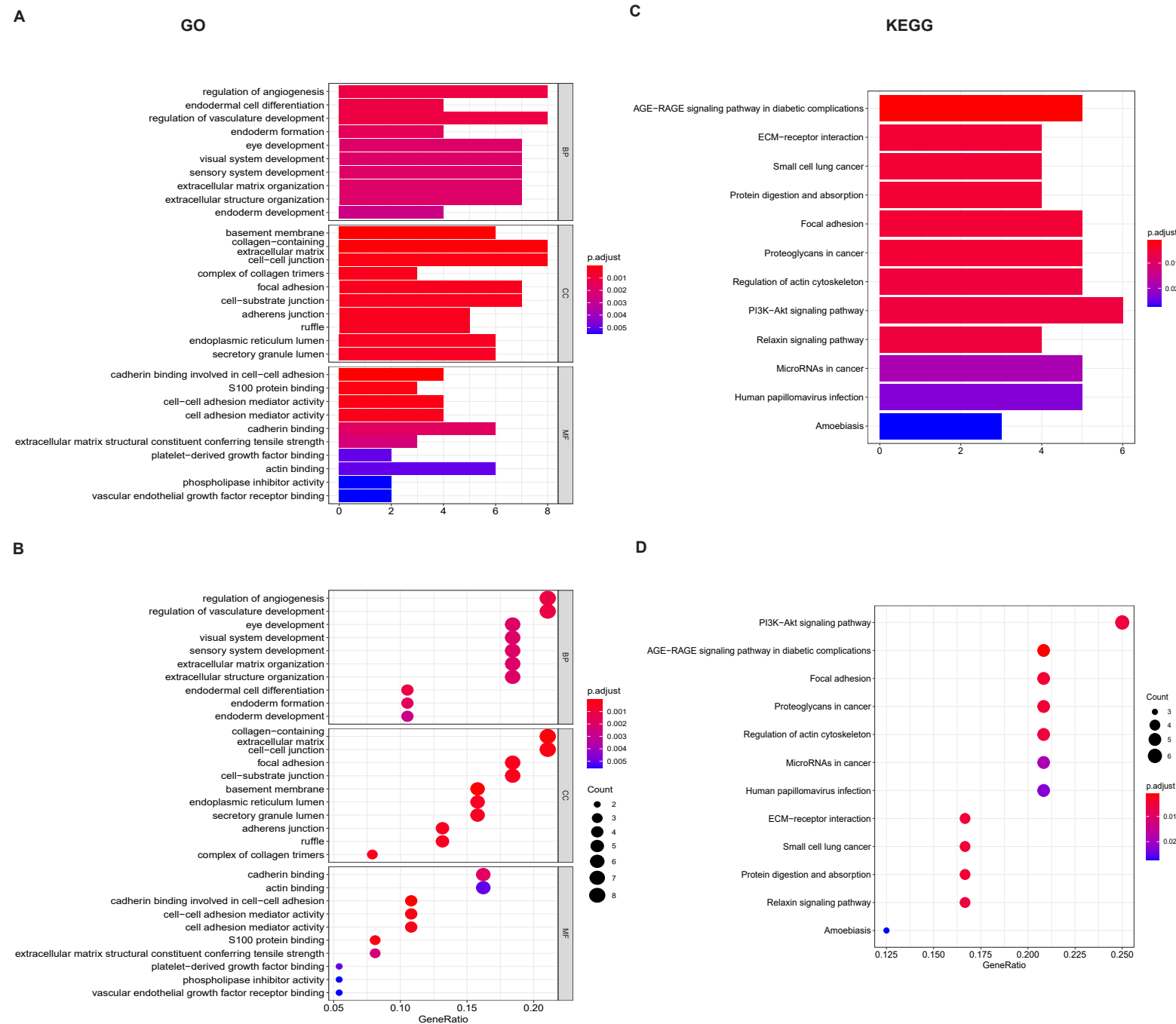

**Figure S5 | GO and KEGG analyses of mRNAs in the ceRNA network. (A, B) GO analysis and (C, D) KEGG analysis.**

Supplement: Supplementary file 2 [file Image5.pdf]
