# Supplementary material for: Identification of Ferroptosis-Related Biomarkers for Prognosis and Immunotherapy in Patients With Glioma
Source: Front Cell Dev Biol. 2022 Jan 31;10:817643. doi: 10.3389/fcell.2022.817643 (PMC8842255; doi:10.3389/fcell.2022.817643)
Supplement: Supplementary file 3 [file Image6.pdf]

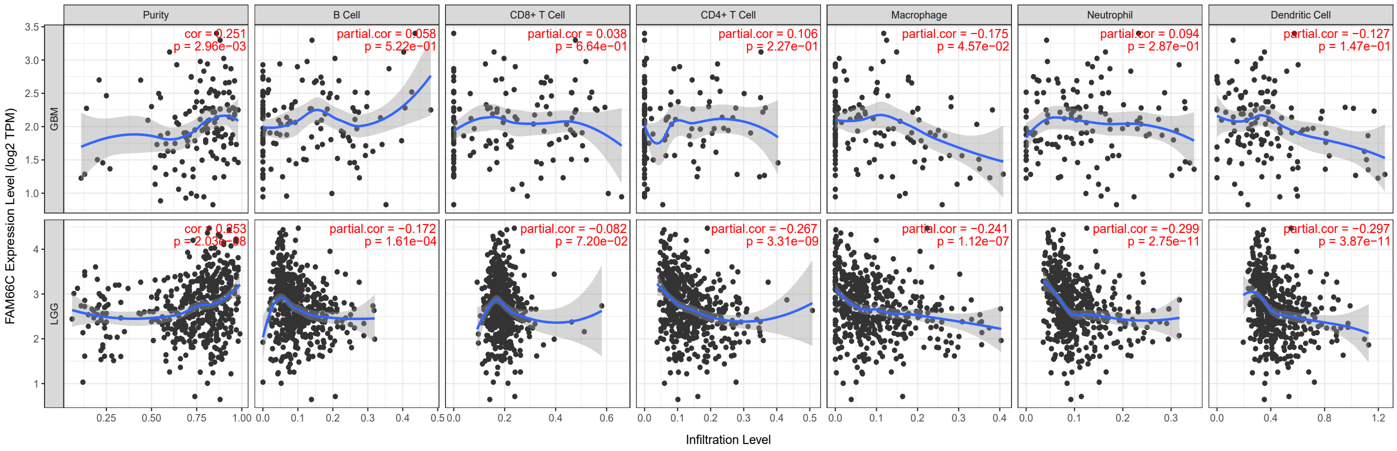

Figure S6 | Correlation analysis between the expression of ferroptosis-related prognostic lncRNA FAM66C and the infiltration of immune cell subtypes from TIMER in glioma.
