# Supplementary material for: Identification of Ferroptosis-Related Biomarkers for Prognosis and Immunotherapy in Patients With Glioma
Source: Front Cell Dev Biol. 2022 Jan 31;10:817643. doi: 10.3389/fcell.2022.817643 (PMC8842255; doi:10.3389/fcell.2022.817643)
Supplement: Supplementary file 4 [file Image4.pdf]

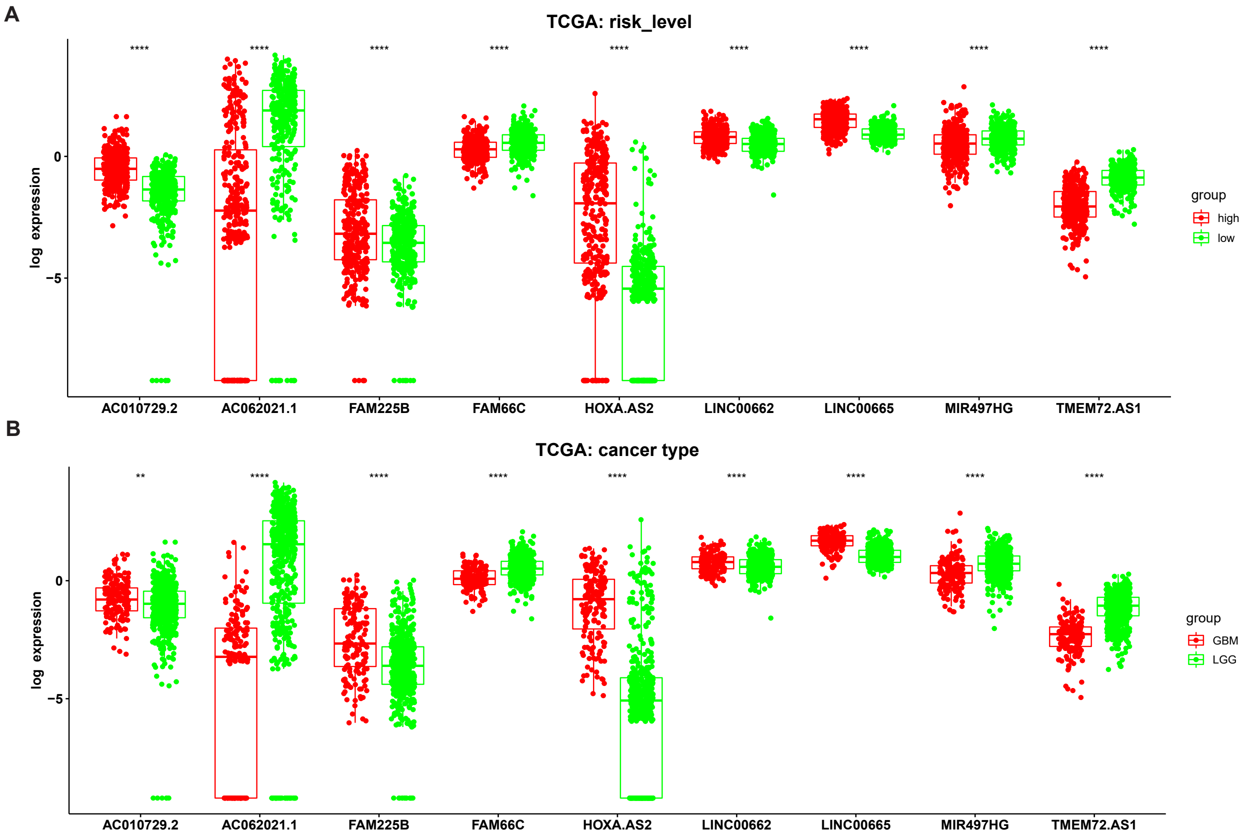

Figure S4 | Correlation between the expression level of 9 ferroptosis-related lncRNAs and clinicopathological features in the validation TCGA cohort. **(A-B)** Risk level and cancer type. GBM: glioblastoma multiform, LGG: Low-grade glioma; *p< 0.05, **p < 0.01, ***p < 0.001.
