# Supplementary material for: Identification of Ferroptosis-Related Biomarkers for Prognosis and Immunotherapy in Patients With Glioma
Source: Front Cell Dev Biol. 2022 Jan 31;10:817643. doi: 10.3389/fcell.2022.817643 (PMC8842255; doi:10.3389/fcell.2022.817643)
Supplement: Supplementary file 5 [file Image2.pdf]

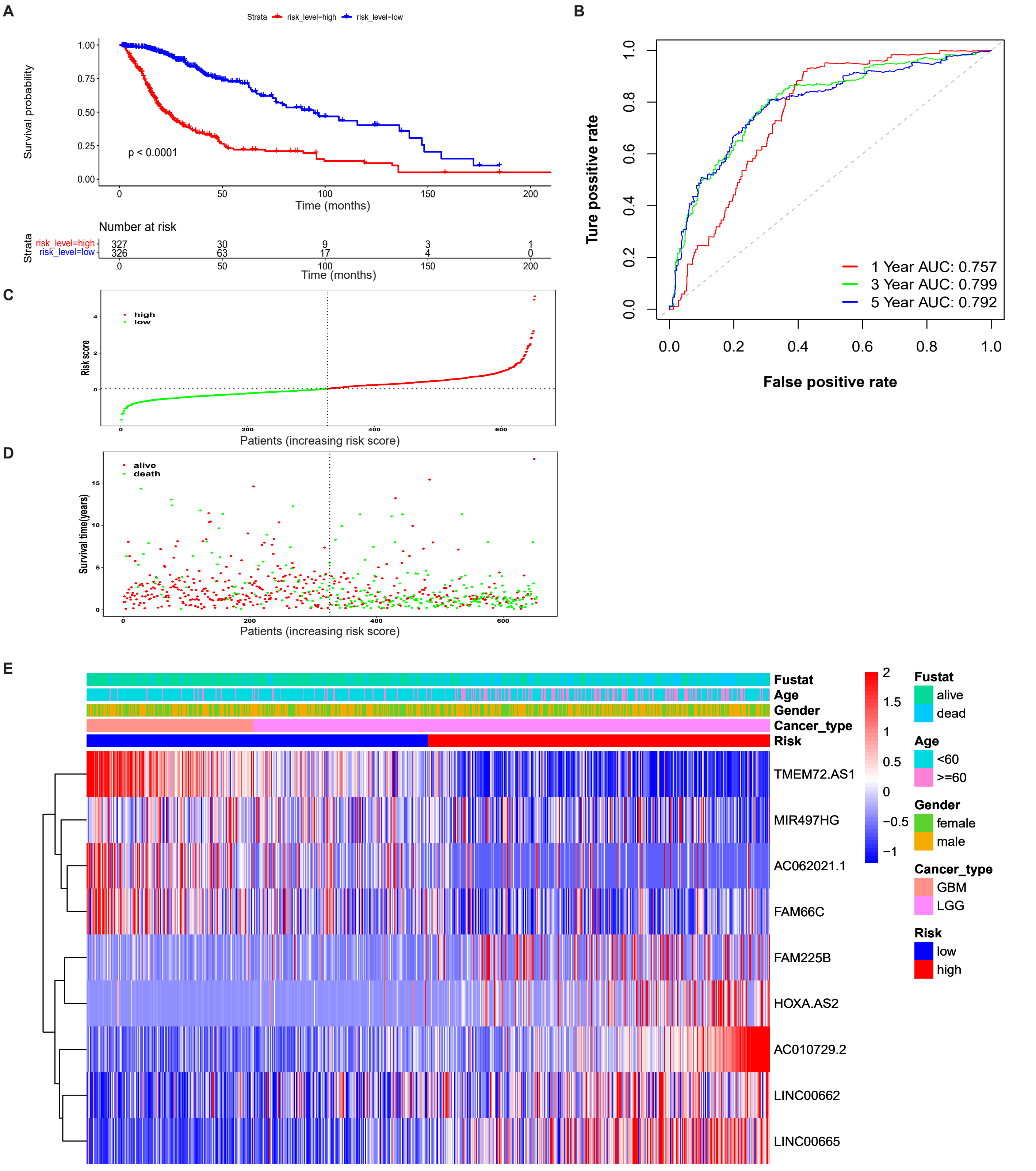

FIGURE S2 | Validation of the risk score model based on ferroptosis-related prognostic biomarkers in the TCGA cohort. **(A)** Kaplan-Meier survival based on risk level. **(B)** ROC curves were used to predict the OS of glioma patients based on risk scores. **(C-E)** The risk score, survival distribution of patients with increased risk scores, and expression heatmap of ferroptosis-related lncRNAs based on risk level.
