# Supplementary material for: Identification of Ferroptosis-Related Biomarkers for Prognosis and Immunotherapy in Patients With Glioma
Source: Front Cell Dev Biol. 2022 Jan 31;10:817643. doi: 10.3389/fcell.2022.817643 (PMC8842255; doi:10.3389/fcell.2022.817643)
Supplement: Supplementary file 6 [file Image3.pdf]

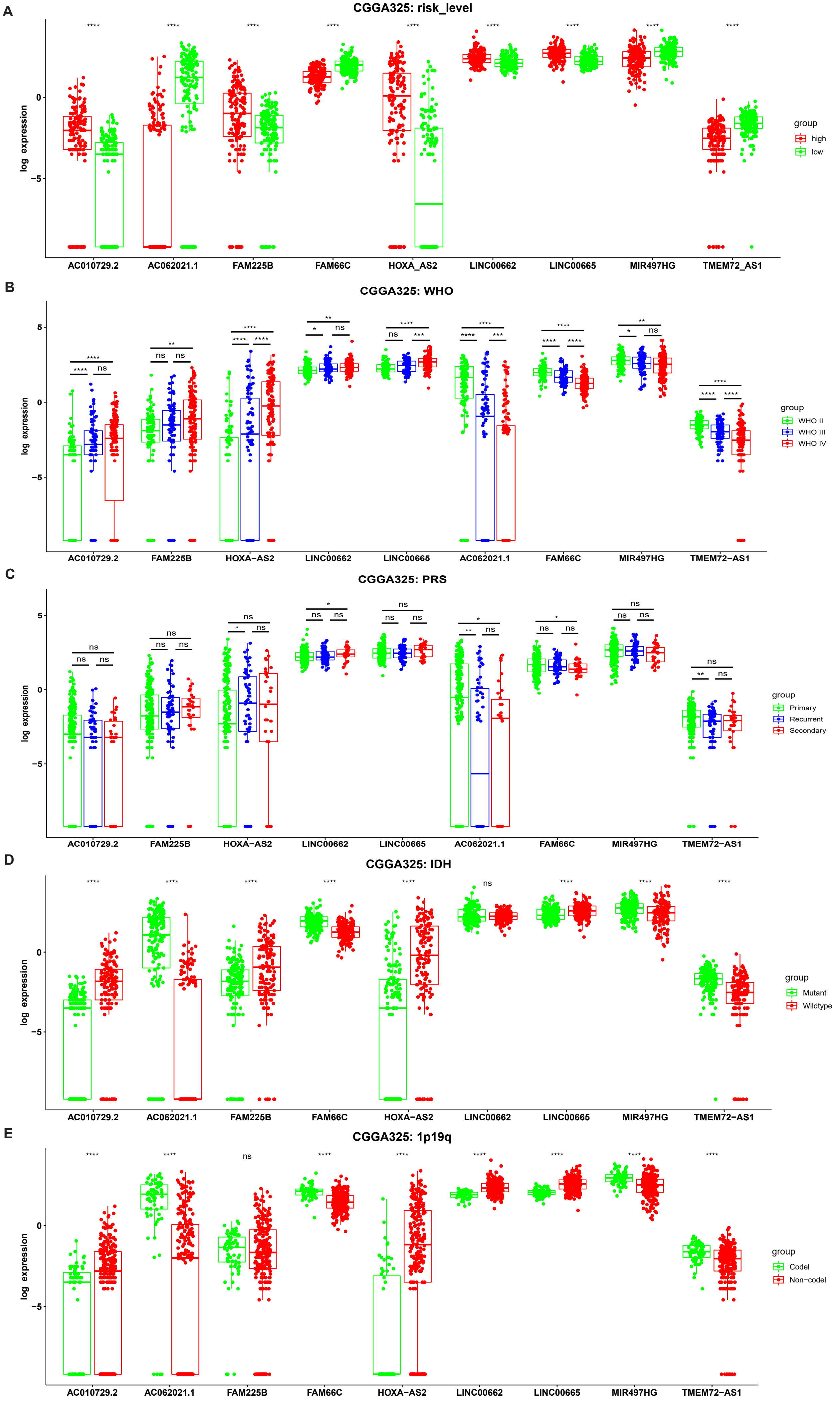

FUGURE S3 | Correlation between the expression level of 9 ferroptosis-related lncRNAs and clinicopathological features in the validation CGGA325 cohort. **(A-E)** Risk level, WHO grade, PRS, IDH mutation status, and 1p19q codeletion status. WHO: World Health Organization; P: Primary, R: Recurrent, S: Secondary Recurrent; ns: Not significant, *$p < 0.05$, **$p < 0.01$, ***$p < 0.001$.
