# Supplementary material for: Identification of Ferroptosis-Related Biomarkers for Prognosis and Immunotherapy in Patients With Glioma
Source: Front Cell Dev Biol. 2022 Jan 31;10:817643. doi: 10.3389/fcell.2022.817643 (PMC8842255; doi:10.3389/fcell.2022.817643)
Supplement: Supplementary file 7 [file Table2.docx]

**Table S2:** Risk score model based on 9 ferroptosis-related prognostic lncRNAs.

| **LncRNAs** | **Cor** | **HR** | **Lower 95%CI** | **Upper 95%CI** | **P Value** |
| --- | --- | --- | --- | --- | --- |
| AC010729.2 | 0.99849102 | 2.714 | 1.831 | 4.024 | 0 |
| AC062021.1 | -0.01261145 | 0.987 | 0.975 | 1 | 0.0457 |
| FAM225B | 0.18957587 | 1.209 | 1.078 | 1.356 | 0.0012 |
| FAM66C | -0.05426328 | 0.947 | 0.903 | 0.994 | 0.0271 |
| HOXA-AS2 | 0.02465331 | 1.025 | 1 | 1.05 | 0.0487 |
| LINC00662 | 0.03022672 | 1.031 | 1.007 | 1.055 | 0.00119 |
| LINC00665 | 0.01995719 | 1.02 | 1.003 | 1.037 | 0.0181 |
| MIR497HG | -0.00924465 | 0.991 | 0.985 | 0.997 | 0.0018 |
| TMEM72-AS1 | -1.09779996 | 0.334 | 0.17 | 0.654 | 0.0014 |

Risk score = β_AC010729.2_ * E_AC010729.2_ + β_AC062021.1_ * E_AC062021.1_ +β_FAM225B_ * E_FAM225B_

+ β_FAM66C_ * E_FAM66C_ + β_AHOXA-AS2_ * E_AHOXA-AS2_ + β_LINC00662_ * E_LINC00662_

+ β_LINC00665_ * E_LINC00665_ + β_MIR497HG_ * E_MIR497HG_ + β_TME72-AS1_ * E_TME72-AS1_
